# Gastrointestinal specific anxiety in irritable bowel syndrome: validation of the Japanese version of the visceral sensitivity index for university students

**DOI:** 10.1186/1751-0759-8-10

**Published:** 2014-03-21

**Authors:** Tatsuo Saigo, Jun Tayama, Toyohiro Hamaguchi, Naoki Nakaya, Tadaaki Tomiie, Peter J Bernick, Motoyori Kanazawa, Jennifer S Labus, Bruce D Naliboff, Susumu Shirabe, Shin Fukudo

**Affiliations:** 1 Center for Health and Community Medicine, Nagasaki University, Nagasaki, Japan; 2 Department of Preventive Medicine, Graduate School of Biomedical Sciences, Nagasaki University, Nagasaki, Japan; 3 Department of Occupational Therapy, School of Health and Social Services, Saitama Prefectural University, Saitama, Japan; 4 Tohoku Medical Megabank Organization, Tohoku University, Miyagi, Japan; 5 Department of Clinical Psychology, School of Psychological Science, Health Sciences University of Hokkaido, Hokkaido, Japan; 6 Department of Behavioral Medicine, Tohoku University Graduate School of Medicine, 2-1 Seiryo, Aoba, Sendai, Miyagi 980-8575, Japan; 7 Department of Medicine, Center for Neurobiology of Stress, David Geffen School of Medicine, University of California Los Angeles, Los Angeles, CA, USA

**Keywords:** Gastrointestinal-specific anxiety, Irritable Bowel Syndrome (IBS), Motility, Psychosomatics, Validation, Visceral Sensitivity Index (VSI)

## Abstract

**Objective:**

The visceral sensitivity index (VSI) is a useful self-report measure of the gastrointestinal symptom-specific anxiety (GSA) of patients with irritable bowel syndrome (IBS). Previous research has shown that worsening GSA in IBS patients is related to the severity of GI symptoms, suggesting that GSA is an important endpoint for intervention. However, there is currently no Japanese version of the VSI. We therefore translated the VSI into Japanese (VSI-J) and verified its reliability and validity.

**Material and methods:**

Participants were 349 university students aged 18 and 19 years and recruited from an academic class. We analyzed data from the VSI-J, Anxiety Sensitivity Index (ASI), Hospital Anxiety and Depression scale (HAD), and Irritable Bowel Syndrome Severity Index (IBS-SI). The internal consistency, stability, and factor structure of the VSI-J and its associations with anxiety, depression and severity measures were investigated.

**Results:**

The factor structure of the VSI-J is unidimensional and similar to that of the original VSI (Cronbach’s α = 0.93). Construct validity was demonstrated by significant correlations with ASI (*r* = 0.43, *p* < 0.0001), HAD-ANX (*r* = 0.19, *p* = 0.0003), and IBS-SI scores (*r* = 0.45, *p* < 0.0001). Furthermore, the VSI-J was a significant predictor of severity scores on the IBS-SI and demonstrated good discriminant (*p* < 0.0001) and incremental (*p* < 0.0001) validity.

**Conclusion:**

These findings suggest that the VSI-J is a reliable and valid measure of visceral sensitivity.

## Introduction

Irritable bowel syndrome (IBS) is a functional gastrointestinal disorder not associated with organic disease that consists of chronic abdominal discomfort associated with abnormal bowel habits [1,2]. The prevalence of IBS has been reported at approximately 5–11% in industrialized countries [3]. Patients with IBS have been shown to have poor health-related quality of life (QOL) when compared with healthy individuals [4] and often experience a significant financial burden—indeed, their economic impact factor increases from 1.1 to 6.0 when compared with non-IBS individuals [5]. The characteristic pathophysiological features of IBS are dysmotility [6,7], visceral sensitivity [8,9], and psychological disturbances [10,11]. The GI symptoms of IBS may aggravate depression and anxiety and be associated with psychosocial stress; indeed, patients with IBS symptoms improve with recovery from psychological disturbances [12-14]. At least two studies have shown that patients with IBS report a high frequency of comorbid anxiety disorders such as panic disorder, agoraphobia, generalized anxiety disorder, and post-traumatic stress disorder [14,15].

Anxiety symptoms play a major role in the onset of IBS and in the maintenance and aggravation of GI symptoms [15]. Further, in IBS, the maintenance and aggravation of GI symptoms and pain is related to brain function through the emotional motor system (EMS) and responses of the neuroendocrine and autonomic nervous systems [16]. For example, in studies that used colonic barostats, the thalamus, insular cortex, anterior cingulate gyrus, and prefrontal cortex were activated; these activations were associated with abdominal pain and psychological abnormalities such as anxiety and depression [12,17-19]. Patients with IBS show greater activation of the prefrontal cortex and anterior cingulate gyrus than do healthy individuals, and also perceive more abdominal pain [12,16-18]. Patients with IBS also demonstrate enhanced pathological vigilance and selective attention for their GI symptoms [20,21]. Thus, the association of brain function with pathological vigilance and selective attention for GI symptoms suggests a neurophysiological foundation for visceral sensitivity [22-24]. GI symptoms are not only external stressors but also act as internal stressors by causing and aggravating anxiety (gastrointestinal-specific anxiety: GSA) [25]. Excessive activity of the amygdala can activate the insular cortex [26], prefrontal cortex, and the anterior cingulate gyrus, which ultimately may lead to GSA and aggravated GI symptoms [4,27]. Interventions aimed at reducing GSA associated with IBS should therefore contribute to an improvement in the QOL of patients with IBS [27,28].

GSA is defined as “the cognitive, affective, and behavioral response stemming from fear of GI sensations, symptoms, and the context in which these visceral sensations and symptoms occur” (28, p.89). For example, the fear of visceral sensations and GI symptoms is related to situations involving restaurants, parties, or first-time visits to locations where bathroom facilities are unknown or difficult to reach; such experiences may cause particular cognitive or behavioral responses. IBS patients may engage in avoidance behaviors that cause GSA, increase vigilance or attention to visceral sensations, and show excessive reactions to low-level visceral sensation [29]. Labus et al. [27] have developed the Visceral Sensitivity Index (VSI) to assess specific GSA seen in patients with IBS.

The validity and reliability of the VSI have been confirmed in several studies (27 28, 30). In studies with both university students and IBS patients, the VSI has been shown to have a unidimensional factor structure [27,28]. A high level of internal consistency has been demonstrated in a combined group of younger healthy participants, IBS patients (Cronbach’s α ranging from 0.90 to 0.92), and adults with IBS (Cronbach’s α = 0.93) [27,28]. Test-retest reliability estimates showed that, four weeks after testing, the inter-class coefficient was 0.86 [30]. Further, the construct validity has been extensively studied; results for the original VSI show moderate to strong positive correlations with measures of anxiety sensitivity (AS), state anxiety, and severity of GI symptoms [27,28,30]. The original VSI also showed a strong positive correlation with the depressive symptoms of IBS patients, but there was no significant correlation with the depressive symptoms of university students with IBS [28]. In addition, the Norwegian version of the VSI showed no correlation with the depressive symptoms or indicators of food sensitivity of IBS patients [30].

There is currently no Japanese version of the VSI; however, there is a clear need for such an instrument because it could be useful for verifying treatment effects in IBS patients and may therefore be indispensable for the future development of IBS treatments [27]. In the present study, we report our newly developed Japanese version of the VSI and test its validity and reliability. Reliability and validity have been confirmed in a previous analog study of university students [28]. As a large sample was necessary to develop the Japanese version of the VSI, the present study was conducted with a sample of university students, including those with symptoms of IBS. We then tested the following hypotheses.

1. The Japanese version of the VSI will exhibit a unidimensional factor structure.

2. The Japanese version of the VSI will show moderate positive correlations with measures of AS, state anxiety, and the severity of IBS symptoms; furthermore, it will show weak positive correlations with measures of depressive symptoms.

3. The Japanese version of the VSI will predict the severity of IBS symptoms after controlling for AS and state anxiety.

## Methods

### Participants

**Figure 1:**
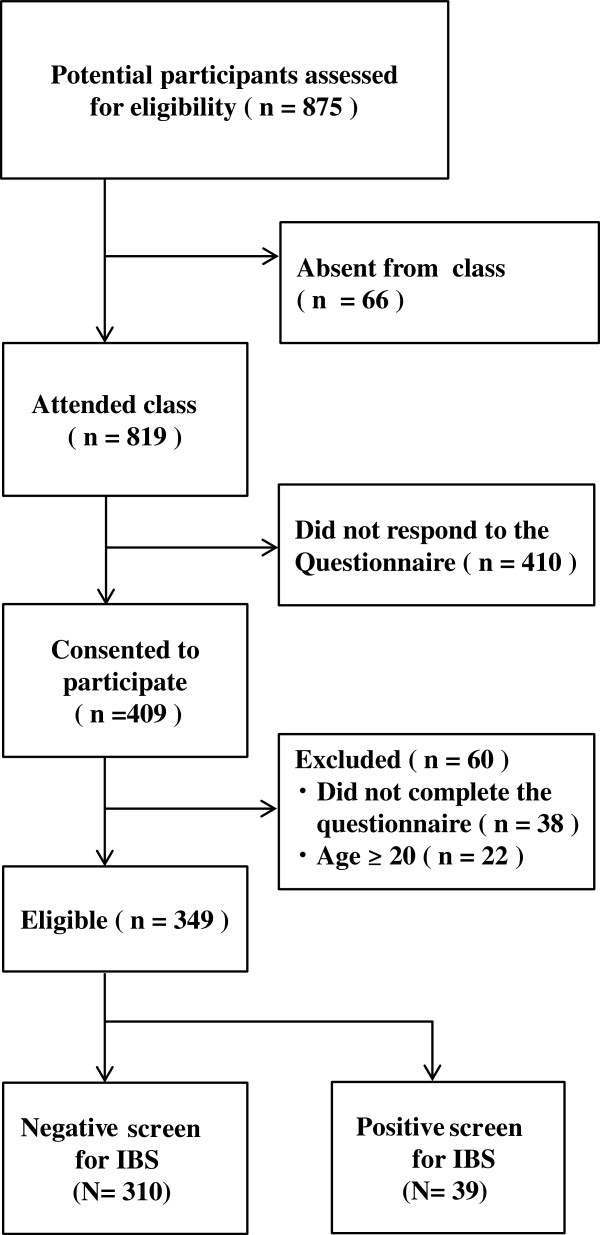
**Flow chart showing the selection of the participants.** Of 875 possible participants, 66 were absent from class, 410 did not respond to the questionnaire, 38 did not fully complete the questionnaire, and 22 were excluded because they were 20 years of age or older. We analyzed the data of the remaining 349 persons who answered all of the items on the questionnaire.

We recruited 875 undergraduate student participants from Nagasaki University in Japan; Figure 1 shows the selection process. For the present study, we were interested in younger participants (less than 20 years old). After screening for age, the data of 349 students (male = 207, female = 142, mean age = 19 years) was available for analysis. The participants answered all items on the VSI questionnaire.

### Japanese Version of the Visceral Sensitivity Index (VSI-J)

The VSI was translated into Japanese using a standardized procedure. Two researchers, one a clinical psychologist knowledgeable in psychosomatic medicine, translated the original version. A medical doctor who specializes in psychosomatic medicine then confirmed the consistency of the translated Japanese version. A bilingual researcher (native English speaker) back-translated the Japanese VSI into English. The VSI-J was established after comparing the results of the back translation to the original, and there was agreement among the researchers about the nuances (Tables 1 and 2).

**Table 1:** Original version of the VSI [27]

**Visceral Sensitivity Index (VSI)**							
**Item**		**Strongly agree**	**Moderately agree**	**Mildly agree**	**Mildly disagree**	**Moderately disagree**	**Strongly disagree**
1	I worry that whenever I eat during the day, bloating and distension in my belly will get worse.	1	2	3	4	5	6
2	I get anxious when I go to a new restaurant.	1	2	3	4	5	6
3	I often worry about problems in my belly.	1	2	3	4	5	6
4	I have a difficult time enjoying myself because I cannot get my mind off of discomfort in my belly.	1	2	3	4	5	6
5	I often fear that I won’t be able to have a normal bowel movement.	1	2	3	4	5	6
6	Because of fear of developing abdominal discomfort, I seldom try new foods.	1	2	3	4	5	6
7	No matter what I eat, I will probably feel uncomfortable.	1	2	3	4	5	6
8	As soon as I feel abdominal discomfort I begin to worry and feel anxious.	1	2	3	4	5	6
9	When I enter a place I haven’t been before, one of the first things I do is to look for a bathroom.	1	2	3	4	5	6
10	I am constantly aware of the feelings I have in my belly.	1	2	3	4	5	6
11	I often feel discomfort in my belly could be a sign of a serious illness.	1	2	3	4	5	6
12	As soon as I awake, I worry that I will have discomfort in my belly during the day.	1	2	3	4	5	6
13	When I feel discomfort in my belly, it frightens me.	1	2	3	4	5	6
14	In stressful situations, my belly bothers me a lot.	1	2	3	4	5	6
15	I constantly think about what is happening inside my belly.	1	2	3	4	5	6

**Table 2:** Japanese version of the VSI

**Visceral Sensitivity Index Japanese version (VSI-J)**							
項目		とてもあてはまる	まあまあ あてはまる	どちらかといえばあてはまる	どちらかといえばあてはまらない	あまりあてはまらない	まったくあてはまらない
1	何かを食べるときは、いつもおなかの膨れる感じや張る感じが悪化することを心配する	1	2	3	4	5	6
2	新しいレストランに行くときは不安になる	1	2	3	4	5	6
3	私はおなかの問題についてよく心配する	1	2	3	4	5	6
4	おなかの不快感が頭から離れないために、楽しく時間を過ごすことが難しい	1	2	3	4	5	6
5	私は排便が正常にならないのではないかとよく恐れている	1	2	3	4	5	6
6	おなかに不快感が生じることを恐れて、新しい食べ物を食べることはほとんどない	1	2	3	4	5	6
7	たとえ何を食べても、おそらく落ち着かない気持ちになる	1	2	3	4	5	6
8	おなかに不快感が生じるとすぐに悩んだり 不安になったりする	1	2	3	4	5	6
9	今まで行ったことがない場所に入った時、まず最初にトイレを探す	1	2	3	4	5	6
10	私はおなかの感覚をいつも意識している	1	2	3	4	5	6
11	おなかの不快感は深刻な病気の兆候ではないかとよく考えてしまう	1	2	3	4	5	6
12	朝起きるとすぐにその日おなかに不快感が生じることを心配する	1	2	3	4	5	6
13	おなかに不快感が生じると怖くなる	1	2	3	4	5	6
14	ストレスの多い状況では、おなかの不快感が増す	1	2	3	4	5	6
15	私は自分のおなかの中で何が起きているかをいつも考える	1	2	3	4	5	6

The VSI measures gastrointestinal specific anxiety using 15 questionnaire items, with responses ranging from 1 (*strongly agree*) to 6 (*strongly disagree*). The VSI was “reverse scored” according to previously established methods [27], with scores ranging from 0 to 5. Total scores ranged from 0 (*no GI-specific anxiety*) to 75 (*severe GI-specific anxiety*).

### Rome III diagnostic questionnaire

Rome III criteria are widely used for the diagnosis of IBS. These criteria include recurrent abdominal pain or discomfort (on at least 3 days/month in the last 3 months) associated with two or more of the following: [1] improved by defecation, [2] onset associated with a change in frequency of stool, and [3] onset associated with a change in form (appearance) of stool. The Rome III diagnostic questionnaire has been confirmed as a reliable and valid self-administered questionnaire for the purpose of diagnosing IBS (Drs. Kanazawa & Fukudo, personal communication). The questionnaire determines whether a person is IBS-positive or non-IBS.

### The IBS Severity Index (IBS-SI)

In the present study, the IBS-SI was used to assess the severity of GI symptoms [31]. The IBS-SI consists of five items with total scores ranging from 0 to 500. Mild, moderate and severe symptoms of IBS are indicated by scores of 75 to 174, 175 to 299, and 300 or over, respectively [31]. The VSI has shown strong positive correlations with the severity of the GI symptoms of both healthy (non-IBS) individuals and individuals with IBS (IBS-positive) [28]. Therefore, the IBS-SI measure of symptom severity was used to examine the construct validity of the VSI-J.

### The Hospital Anxiety and Depression scale (HAD)

The HAD is a 14-item self-administered anxiety and depression scale specifically designed for use in non-psychiatric settings [32]. Seven of the items relate to anxiety (HAD-ANX) and seven to depression (HAD-DEP). Participants rate each item on a scale from 0 to 3. Higher ratings indicate a greater number of symptoms of anxiety or depression. Two scores (one each for the HAD-ANX and the HAD-DEP) are calculated, each ranging from 0 to 21. Scores of 0 to 7 on the respective subscales are considered normal, with 8 to 10 being borderline and scores of 11 to 21 indicating probable clinical cases [32]. Our scoring methods conformed to those of the original version of the scale [32]. Scores on the original VSI were significantly and moderately correlated with scores on the HAD-ANX but showed a weak correlation with HAD-DEP scores [28]. Therefore, the HAD was used to examine the construct validity of the VSI-J.

### The Anxiety Sensitivity Index (ASI)

The ASI is a 16-item self-administered questionnaire about anxiety sensitivity designed to assess fear of bodily sensations associated with arousal [33,34]. Items are rated on 5-point Likert scales ranging from 0 (*very little*) to 4 (*very much*). Previous research has shown that the original VSI is significantly and strongly correlated with measures of AS among both IBS patients and healthy individuals [28]. AS was related to an understanding of GSA constructed through introspection and resulting in worry, fear, and vigilance [27,28]. Therefore, the ASI was used to examine the construct validity of the VSI-J.

### Procedure

The questionnaire survey was presented in the classroom during an academic session. We explained the research procedures and general purpose, and provided both a written and verbal explanation of the intended use of the data. In addition, we explained that persons who did not consent to participate in the study would be placed at no disadvantage whatsoever. We only analyzed the data of those students who agreed to participate.

### Statistical analysis

Analyses were carried out using IBM SPSS Statistics 20.0 (SPSS, Inc., Chicago, IL, USA) Descriptive statistics are presented as means ± standard deviations (SD), along with 95% confidence intervals. We used unpaired *t*-tests to compare IBS-positive and non-IBS groups. The factor structure of the VSI-J was confirmed by exploratory factor analysis (EFA). The internal consistency reliability of the VSI-J was determined using Cronbach’s α. The construct validity was measured using Pearson’s correlation coefficients between the VSI-J and scores on the ASI, HAD-ANX, HAD-DEP, and IBS-SI. The discriminant validity of the VSI-J was measured using the partial correlation coefficient between the VSI-J and the IBS-SI while controlling for HAD-ANX and ASI scores. Hierarchical multiple regression analyses were conducted to examine the incremental validity of the VSI-J; IBS-SI scores were the dependent variable and the scores on the VSI-J, ASI, and HAD-ANX were independent variables. For all tests, a *p*-value of .05 (two-tailed) was regarded as the cutoff for statistical significance.

### Ethics

The study protocol was approved by the Ethics Committee of Nagasaki University, which confirmed that the study design was in accordance with the Declaration of Helsinki; all participants provided informed consent.

## Results

### Demographics

Table 3 shows the demographic data for the present study participants, and reference data for university students obtained in a previous study [28]. The prevalence of IBS in the study sample was 11%; the overall gender distribution of the sample was 59% male and 41% female. However, within the IBS-positive group, 67% were female and 33% were male. The scores were significantly higher for the IBS-positive group than for the non-IBS group on the VSI-J (total; *t* = 4.96, *p* < 0.0001), the ASI (*t* = 2.86, *p* = 0.0044), and the IBS-SI (*t* = 8.59, *p* < 0.0001). However, there was no significant difference in the total HAD-ANX (*t* = 1.20, *p* = 0.2305) and total HAD-DEP (*t* = 0.26, *p* = 0.7931) scores.

**Table 3:** Age, sex, and inventory scores

**Variables**	**All participants**	**Non-IBS**	**IBS+**	**Reference value [**[28]**]**	
	**(*N *** **= 349)**	**(*N *** **= 310)**	**(*N *** **= 39)**	**Non-IBS**	**IBS+**
Age	18.8 ± 0.5 [18.7–18.8]	18.8 ± 0.6 [18.7–18.9]	18.8 ± 0.6 (18.6–18.8)	19	19
Sex (% male)	59 [54–64]	63 [57–68]	33 [21–49]	-	-
IBS + (%)	11 [8–15]	-	-	-	-
VSI	15.2 ± 13.6 [13.8–16.6]	14.0 ± 12.9 [12.5–15.4]	25.1 ± 15.3 [20.2–30.1]^**^	8.2 [7.2–9.2]	28.0 [22.1–33.9]
ASI	16.6 ± 9.7 [15.7–17.6]	16.1 ± 9.2 [15.0–17.1]	20.8 ± 12.4 [16.7–24.8]^**^	18.6 [18.5–18.7]	23.2 [19.5–26.9]
HAD-ANX	8.5 ± 4.0 [8.1–8.9]	8.4 ± 4.0 [8.0–8.9]	9.3 ± 4.5 [7.8–10.7]	6.5 [6.1–6.9]	8.5 [7.7–9.3]
HAD-DEP	7.6 ± 3.1 [7.3–7.9]	7.6 ± 3.1 [7.3–8.0]	7.5 ± 2.9 [6.5–8.4]	3.1 [2.7–3.5]	5.4 [5.0–5.8]
IBS-SI	103.8 ± 86.7 [96.1–114.0]	90.9 ± 77.3 [82.3–99.6]	206.2 ± 91.0 [176.7–225.7]^**^	-	-

Data are expressed as: mean ± standard deviation [95% confidence interval].

*Abbreviations*: *VSI* Visceral Sensitivity Index, *ASI* Anxiety Sensitivity Index, *HAD-ANX* and *HAD-DEP* subscales of the Hospital Anxiety and Depression Scale, *IBS-SI* Irritable Bowel Syndrome Severity Index, *IBS +* positive diagnosis of Irritable Bowel Syndrome, *Non-IBS* no presence of Irritable Bowel Syndrome.

**p < 0.01 for IBS + vs. Non-IBS.

### Factor structure

To examine the factor structure of the VSI-J, we conducted an EFA (using maximum likelihood estimation and promax rotation) without assigning the number of factors (Table 4). There are four criteria for estimating factor structure: [1] initial eigenvalues greater than one, [2] shape of the scree plot, [3] interpretations of factor structure, and [4] factor loadings greater than 0.40. Initial eigenvalues greater than one were observed for two factors (first factor eigenvalue = 8.18, second factor eigenvalue = 1.17). However, the shape of the scree plot suggested a unidimensional factor structure. Accordingly, we then analyzed the factor structure by conducting exploratory analyses and assigning either one or two factors. The results confirmed a unidimensional factor structure. That is, a single factor accounted for 54% of the total variance. This was consistent with findings for the original VSI [27,28].

**Table 4:** Factor analysis of the Japanese version of the Visceral Sensitivity Index

**Item**	**Statement**	**Factor loadings**	**Item-total correlation**
1	I worry that whenever I eat during the day, bloating and distension in my belly will get worse.	0.53	0.61
2	I get anxious when I go to a new restaurant.	0.42	0.52
3	I often worry about problems in my belly.	0.72	0.78
4	I have a difficult time enjoying myself because I cannot get my mind off of discomfort in my belly.	0.74	0.79
5	I often fear that I won’t be able to have a normal bowel movement.	0.77	0.79
6	Because of fear of developing abdominal discomfort, I seldom try new foods.	0.56	0.62
7	No matter what I eat, I will probably feel uncomfortable.	0.62	0.66
8	As soon as I feel abdominal discomfort I begin to worry and feel anxious.	0.82	0.82
9	When I enter a place I haven’t been before, one of the first things I do is to look for a bathroom.	0.58	0.62
10	I am constantly aware of the feelings I have in my belly.	0.83	0.82
11	I often feel discomfort in my belly could be a sign of a serious illness.	0.81	0.80
12	As soon as I awake, I worry that I will have discomfort in my belly during the day.	0.82	0.81
13	When I feel discomfort in my belly, it frightens me.	0.82	0.82
14	In stressful situations, my belly bothers me a lot.	0.68	0.72
15	I constantly think about what is happening inside my belly.	0.81	0.80
Eigenvalue		8.18	
Variance (%)		54.56	

Note. While factor analysis was performed with the Japanese version of the VSI, the original English items are given here for ease of reference.

### Reliability

To examine reliability, we calculated Cronbach’s α for all scale measures. All measures demonstrated high internal consistency and were therefore considered reliable (VSI-J, α = 0.93; ASI, α = 0.88; total HAD, α = 0.73; HAD-ANX, α = 0.66; HAD-DEP, α = 0.50; IBS-SI; α = 0.74).

### Validity

#### Construct validity

Table 5 gives Pearson’s correlation coefficients among the VSI-J, ASI, HAD-ANX, HAD-DEP and IBS-SI. Overall, scores on the VSI-J were significantly correlated with scores on the ASI (*r* = 0.46, *p* < 0.0001), HAD-ANX (*r* = 0.19, *p* = 0.0003), and IBS-SI (*r* = 0.43, *p* < 0.0001). However, there was no significant correlation with the HAD-DEP (*r* = 0.10, *p* = 0.0511). For the IBS-positive group (n = 39), there was a significant correlation between scores on the VSI-J and scores on the ASI (*r* = 0.44, *p* = 0.0048), HAD-ANX (*r* = 0.32, *p* = 0.0445), and IBS-SI (*r* = 0.65, *p* < 0.0001). However, there was no significant correlation between VSI-J and HAD-DEP scores (*r* = 0.27, *p* = 0.0976). For the non-IBS group (n = 310), there was a significant correlation between scores on the VSI-J and scores on the ASI (*r* = 0.41, *p* < 0.0001), HAD-ANX (*r* = 0.16, *p* = 0.0051), and IBS-SI (*r* = 0.36, *p* < 0.0001). However, there was no significant correlation between VSI-J and HAD-DEP scores (*r* = 0.09, *p* = 0.1096).

**Table 5:** Correlation matrices for the construct validation of the Japanese version of the VSI

	**VSI**	**ASI**	**HAD-ANX**	**HAD-DEP**
**All (*N *** **= 349)**				
ASI	0.46^**^	-	-	-
HAD-ANX	0.19^**^	0.30^**^	-	-
HAD-DEP	0.10	0.17^**^	0.53^**^	-
IBS-SI	0.43^**^	0.24^*^	0.09	0.09
**IBS + (*N *** **= 39)**				
ASI	0.44^**^	-	-	-
HAD-ANX	0.32^*^	0.57^**^	-	-
HAD-DEP	0.27	0.47^**^	0.60^**^	-
IBS-SI	0.65^**^	0.50^**^	0.30	0.24
**Non-IBS (*N *** **= 310)**				
ASI	0.41^**^	-	-	-
HAD-ANX	0.16^**^	0.24^**^	-	-
HAD-DEP	0.09	0.13^*^	0.52^**^	-
IBS-SI	0.36^**^	0.14^*^	0.04	0.09

*Abbreviations*: *VSI* Visceral Sensitivity Index, *ASI* Anxiety Sensitivity Index, *HAD-ANX* and *HAD-DEP* subscales of the Hospital Anxiety and Depression scale, *IBS-SI* Irritable Bowel Syndrome Severity Index.

*p < 0.05, **p < 0.01.

**Table 6:** Hierarchical multiple regression analysis with IBS-SI as the dependent variable

**Variable**	**Step 1**				**Step 2**			
	**B**	**SE B**	***β***	***t *** **value**	**B**	**SE B**	***β***	***t *** **value**
Step 1								
HAD-ANX	0.52	1.16	0.02	0.44	−0.13	1.16	0.01	0.12
ASI	2.06	0.49	0.23	4.22^**^	0.45	0.49	0.05	0.93
Step 2								
VSI					2.78	0.33	0.43	8.23^**^
*F* value	10.51^**^				30.94^**^			
Adj *R*^2^	0.052				0.205			
Δ*R*^2^	0.06^**^				0.16^**^			

Note. *N* = 349.

*Abbreviations*: *IBS-SI* Irritable Bowel Syndrome Severity Index, *B* regression coefficients, *SE B* standard errors for regression coefficients, *β* standard regression coefficient, *Adj R*^*2*^ adjusted multiple correlation coefficient, Δ*R*^2^ changes in the multiple squared correlation coefficient, VSI = Visceral Sensitivity Index, *ASI* Anxiety Sensitivity Index, *HAD-ANX* subscale of the Hospital Anxiety and Depression scale.

**p < 0.01.

#### Discriminant validity

To examine the discriminant validity of the VSI-J, we calculated the partial correlation coefficient (for the whole sample, *N* = 349) between the VSI-J and IBS-SI while controlling for scores on the HAD-ANX and ASI. The result was a significant correlation between scores on the VSI-J and the IBS-SI (*r* = 0.45, *p* < 0.0001). Furthermore, there was a significant correlation between VSI-J and IBS-SI scores when controlling for only the ASI (*r* = 0.40, *p* < 0.0001).

#### Incremental validity

To examine the ability of the VSI-J to predict GI symptoms, we conducted a hierarchical multiple regression analysis (for the whole sample, *N* = 349) with the IBS-SI score as the dependent variable and the VSI-J, ASI, and HAD-ANX scores as independent variables (Table 6). For the first step, independent variables were the ASI and HAD-ANX scores; for the second step, the independent variable was the VSI-J score. The adjusted multiple correlation coefficient for the IBS-SI increased significantly from the first to the second step (5.2% to 20.5%). These findings suggest that the VSI-J contributed unique effects to the IBS-SI.

## Discussion

Our hypotheses were mainly supported by the findings of the present study. As expected, the VSI-J exhibited a unidimensional factor structure (Hypothesis 1). Further, VSI-J scores were significantly and moderately correlated with scores on the ASI, HAD-ANX, and IBS-SI (Hypothesis 2); however, there was no significant correlation between the VSI-J scores and those of the HAD-DEP. As anticipated, when we controlled for ASI (anxiety sensitivity) and HAD-ANX (anxiety) scores, scores on the VSI-J predicted IBS-SI (GI symptom severity; Hypothesis 3).

Our study was able to demonstrate that the VSI-J is a sufficiently reliable measure of anxiety about GI symptoms. We demonstrated (using EFA) that the index has a unidimensional factor structure similar to that of the original VSI. Cronbach’s α was similar to that found in previous studies [27,28] with the original VSI (α = 0.93 vs. α = 0.90–0.93, respectively), indicating that the VSI-J also has high levels of internal consistency.

We were also able to verify the construct validity of the VSI-J. Correlational analyses showed that scores on the VSI-J were related to scores on measures of anxiety sensitivity (ASI), anxiety (HAD-ANX), and the severity of symptoms (IBS-SI). This is consistent with previous research with the original VSI [27,28]. We found a significant correlation between measures of anxiety sensitivity and gastrointestinal specific anxiety as measured by the VSI-J, confirming previous studies showing that individuals with high trait anxiety and high anxiety sensitivity are more likely to report feeling anxious about their gastrointestinal symptoms [27,28].

Generally, in IBS, a correlation is seen between depression and anxiety [11]. It has also been reported that as GI symptoms increase in severity, psychological distress also increases [12-14]. A previous study has reported that scores on the VSI and the HAD-DEP are significantly correlated in patients with a clinical diagnosis of IBS, although this correlation was not observed in university students with symptoms of IBS [28]. Given previously reported findings regarding the correlation between depression and anxiety in individuals diagnosed with IBS, we expected to see at least a weak correlation between the VSI-J scores and the scores on the depression measure (HAD-DEP). However, we did not find such a correlation. This may be due to the fact that, of participants in this study, those with IBS symptoms only experienced moderately severe GI symptoms, as measured by the IBS-SI.

Our analyses revealed two findings important for understanding the relationship between anxiety and gastrointestinal symptoms. The results of the partial correlation analysis showed that the VSI-J has discriminant validity, as shown by a significant correlation between the VSI-J and the severity measure (IBS-SI) independent of the effects of anxiety and anxiety sensitivity. Further, the results of the hierarchical multiple regression analysis showed that the VSI-J has incremental validity that uniquely affects the IBS-SI. Therefore, it may be appropriate to assess the symptoms of the IBS patient by treating GSA rather than anxiety or AS. Together, these results suggest two intriguing possibilities. First, AS is highly relevant in cases of respiratory and cardiac sensation [35]. It is therefore possible that the VSI-J measures fear responses to GI symptoms and GI sensations, and that it is possible to predict the severity of IBS-SI scores. Second, hyperactivity in the hypothalamic-pituitary-adrenal axis and prefrontal cortex is associated with increased levels of visceral sensitivity, which may exacerbate GI symptoms [36]. Consequently, it is possible that GSA is associated with symptom severity because of the direct path of the efferent nerve originating in the brain and projecting to the gastrointestinal tract.

It is desirable to conduct additional studies to further confirm the reliability and validity of the VSI-J, and to address the following points.

First, this study included only students attending a university located in a medium-sized city on the island of Kyushu in western Japan. Additionally, the present study was based solely on results from a screening questionnaire; it did not include diagnosis or evaluation of symptom severity by a physician. Therefore, it is not known whether these results can be extended to clinical samples in the general population. It will be important to replicate our findings with a general population of patients diagnosed with IBS. At the same time, however, the prevalence of IBS in this study (14%) was similar to that reported for the general population in Japan [37]. It has also been reported that individuals who had never sought treatment for IBS symptoms had higher state anxiety compared with healthy subjects, which matches the pattern usually seen in IBS patients [37]. Thus, we believe sampling bias was unlikely to have affected the conclusions of the present study.

Additionally, at this time, the total VSI-J score does not distinguish between minor and severe symptoms. In the future, it will be necessary to set guidelines and cut-off points so that the scale may be used in clinical settings.

Finally, attention to the GI symptoms of IBS patients may be related to activity in the anterior cingulate and prefrontal cortex [38]. It is important to further study the relationship between executive functioning and GSA, so that eventually the VSI may be used as a measure of brain-gut interaction.

In terms of clinical application, reductions in visceral sensitivity (as measured by the VSI-J) are anticipated as a possible endpoint for IBS interventions, which incorporate pharmacotherapy, exercise therapy, psychotherapy, and nutritional therapy. In recent years, the VSI has been used in Western countries when implementing cognitive behavioral therapy to reduce anxiety associated with IBS symptoms [29,39,40]. Cognitive behavioral therapy is used with the goal of changing individuals’ evaluations of threat in situations that cause symptom anxiety and to reduce avoidance behavior. This approach has been shown to significantly improve GI symptoms and GSA [29].

In conclusion, the present study examined the reliability and validity of the newly developed VSI-J in a sample of university students. The scale was found to be reliable and valid in a manner consistent with the original VSI. The VSI-J may now be used to better assess the impact of interventions targeting the reduction of GSA in Japanese patients with IBS. Furthermore, the results of this study may aid in efforts to use the VSI to compare psychological aspects of IBS patients across cultures.

## Competing interests

The authors report no conflicts of interests.

## Authors’ contributions

TS and JT participated in the design of this study, carried out data collection, translated VSI into Japanese, analyzed the data and drafted the manuscript. TH, NN and TT provided the information about the psychological variables. PB carried out back translation into English, and confirmed phrasing nuances with JL and BN. MK, SS and SF evaluated the results of the study and reviewed the manuscript. All authors read and approved the final manuscript.

## References

[B1] Drossman DA The functional gastrointestinal disorders and the Rome III process Gastroenterology 2006 8 1377 1390 10.1053/j.gastro.2006.03.008 16678553

[B2] Longstreth GF Thompson WG Chey WD Houghton LA Mearin F Spiller RC Functional bowel disorders Gastroenterology 2006 8 1480 1491 10.1053/j.gastro.2005.11.061 16678561

[B3] Spiller R Aziz Q Creed F Emmanuel A Houghton L Hungin P Jones R Kumar D Rubin G Trudgill N Whorwell P Guidelines on the irritable bowel syndrome: mechanisms and practical management Gut 2007 8 1770 1798 10.1136/gut.2007.119446 17488783 PMC2095723

[B4] Jerndal P Ringström G Agerforz P Karpefors M Akkermans LM Bayati A Simrén M Gastrointestinal-specific anxiety: an important factor for severity of GI symptoms and quality of life in IBS Neurogastroenterol Motil 2010 8 646 e179 10.1111/j.1365-2982.2010.01493.x 20367800

[B5] Maxion-Bergemann S Thielecke F Abel F Bergemann R Costs of irritable bowel syndrome in the UK and US Pharmacoeconomics 2006 8 21 37 10.2165/00019053-200624010-00002 16445300

[B6] Kellow JE Phillips SF Altered small bowel motility in irritable bowel syndrome is correlated with symptoms Gastroenterology 1987 8 1885 1893 3569764 10.1016/0016-5085(87)90620-2

[B7] Drossman DA Sandler RS McKee DC Levity AJ Bowel patterns among subjects not seeking health care Gastroenterology 1982 8 529 532 7095360

[B8] Mayer EA Raybould HE Role of visceral afferent mechanism in functional bowel disorders Gastroenterology 1987 8 1688 1704 10.1016/0016-5085(90)90475-g 2227282

[B9] Fukudo S Kanazawa M Kano M Sagami Y Endo Y Utsumi A Nomura T Hongo M Exaggerated motility of the descending colon with repetitive distention of the sigmoid colon in patients with irritable bowel syndrome J Gastroenterol 2002 8 suppl 14 145 150 12572883 10.1007/BF03326434

[B10] Drossman DA McKee DC Sandler RS Mitchell CM Cramer EM Lowman BC Burger AL Psychosocial factors in the irritable bowel syndrome: a multivariate study of patients and nonpatients with irritable bowel syndrome Gastroenterology 1988 8 701 708 3396817 10.1016/s0016-5085(88)80017-9

[B11] Whitehead WE Bosmajian L Zonderman AB Costa PT Schuster MM Symptoms of psychologic distress associated with irritable bowel syndrome: comparison of community and medical clinic samples Gastroenterology 1988 8 709 714 3396818 10.1016/s0016-5085(88)80018-0

[B12] Mertz H Morgan V Tanner G Pickens D Price R Shyr Y Kessler R Regional cerebral activation in irritable bowel syndrome and control subjects with painful and nonpainful rectal distention Gastroenterology 2000 8 842 848 10.1016/S0016-5085(00)70170-3 10784583

[B13] Fukudo S Nomura T Muranaka M Taguchi F Brain-gut response to stress and cholinergic stimulation in irritable bowel syndrome: a preliminary study J Clin Gastroenterol 1993 8 133 141 10.1097/00004836-199309000-00009 8031340

[B14] Lydiard RB Irritable bowel syndrome, anxiety, and depression: what are the links? J Clin Psychiatry 2001 8 38 47 12108820

[B15] Sykes MA Blanchard EB Lackner J Keefer L Krasner S Psychopathology in irritable bowel syndrome: support for a psychophysiological model J Behav Med 2003 8 361 372 10.1023/A:1024209111909 12921009

[B16] Mayer EA The neurobiology of stress and gastrointestinal disease Gut 2000 8 861 869 10.1136/gut.47.6.861 11076888 PMC1728136

[B17] Drossman DA Ringel Y Vogt BA Leserman J Lin W Smith JK Whitehead W Alterations of brain activity associated with resolution of emotional distress and pain in a case of severe irritable bowel syndrome Gastroenterology 2003 8 754 761 10.1053/gast.2003.50103 12612913

[B18] Silverman DH Munakata JA Ennes H Mandelkern MA Hoh CK Mayer EA Regional cerebral activity in normal and pathological perception of visceral pain Gastroenterology 1997 8 64 72 10.1016/S0016-5085(97)70220-8 8978344

[B19] Naliboff BD Derbyshire SW Munakata J Berman S Mandelkern M Chang L Mayer EA Cerebral activation in patients with irritable bowel syndrome and control subjects during rectosigmoid stimulation Psychosom Med 2001 8 365 375 11382264 10.1097/00006842-200105000-00006

[B20] Munakata J Naliboff BD Harraf F Kodner A Lembo T Chang L Silverman DH Mayer EA Repetitive sigmoid stimulation induces rectal hyperalgesia in patients with irritable bowel syndrome Gastroenterology 1997 8 55 63 10.1016/S0016-5085(97)70219-1 8978343

[B21] Keough ME Timpano KR Zawilinski LL Schmidt NB The association between irritable bowel syndrome and the anxiety vulnerability factors: body vigilance and discomfort intolerance J Health Psychol 2011 8 91 98 10.1177/1359105310367689 20631041

[B22] Yágüez L Coen S Gregory LJ Amaro E Altman C Brammer MJ Bullmore ET Williams SC Aziz Q Brain response to visceral aversive conditioning: a functional magnetic resonance imaging study Gastroenterology 2006 8 1819 1829 10.1053/j.gastro.2005.02.068 15940617

[B23] Kanazawa M Endo M Yamaguchi K Hamaguchi T Whitehead WE Itoh M Fukudo S Classical conditioned response of rectosigmoid motility and regional cerebral activity in humans Neurogastroenterol Motil 2005 8 705 713 10.1111/j.1365-2982.2005.00691.x 16185309

[B24] Labus JS Naliboff BD Berman SM Suyenobu B Vianna EP Tillisch K Mayer EA Brain networks underlying perceptual habituation to repeated aversive visceral stimuli in patients with irritable bowel syndrome Neuroimage 2009 8 952 960 10.1016/j.neuroimage.2009.05.078 19501173 PMC3399695

[B25] Mayer EA Naliboff BD Chang L Coutinho SV Stress and the gastrointestinal tract: V. stress and irritable bowel syndrome Am J Physiol Gastrointest Liver Physiol 2001 8 G519 G524 11254476 10.1152/ajpgi.2001.280.4.G519

[B26] Wilder-Smith CH Schindler D Lovblad K Redmond SM Nirkko A Brain functional magnetic resonance imaging of rectal pain and activation of endogenous inhibitory mechanisms in irritable bowel syndrome patient subgroups and healthy controls Gut 2004 8 1595 1601 10.1136/gut.2003.028514 15479679 PMC1774298

[B27] Labus JS Bolus R Chang L Wiklund I Naesdal J Mayer EA Naliboff BD The visceral sensitivity index: development and validation of a gastrointestinal symptom-specific anxiety scale Aliment Pharmacol Ther 2004 8 89 97 15225175 10.1111/j.1365-2036.2004.02007.x

[B28] Labus JS Mayer EA Chang L Bolus R Naliboff BD The central role of gastrointestinal-specific anxiety in irritable bowel syndrome: further validation of the visceral sensitivity index Psychosom Med 2007 8 89 98 10.1097/PSY.0b013e31802e2f24 17244851

[B29] Craske MG Wolitzky-Taylor KB Labus J Wu S Frese M Mayer EA Naliboff BD A cognitive-behavioral treatment for irritable bowel syndrome using interoceptive exposure to visceral sensations Behav Res Ther 2011 8 413 421 10.1016/j.brat.2011.04.001 21565328 PMC3100429

[B30] Lind R Lied GA Lillestøl K Valeur J Berstad A Do psychological factors predict symptom severity in patients with subjective food hypersensitivity? Scand J Gastroenterol 2010 8 835 843 10.3109/00365521003797213 20433401

[B31] Shinozaki M Kanazawa M Sagami Y Endo Y Hongo M Drossman DA Whitehead WE Fukudo S Validation of the Japanese version of the Rome II modular questionnaire and irritable bowel syndrome severity index J Gastroenterol 2006 8 491 494 10.1007/s00535-006-1799-9 16799892

[B32] Zigmond AS Snaith PP The hospital anxiety and depression scale Acta Psychiatr Scand 1983 8 361 370 10.1111/j.1600-0447.1983.tb09716.x 6880820

[B33] Reiss S Peterson RA Taylor S Schmidt N Weems CF Anxiety sensitivity index consolidated user manual: ASI, ASI-3, and CASI 2008 Ohio: IDS Publishing Corporation

[B34] Reiss S Peterson RA Gursky DM Mcnally RJ Anxiety sensitivity, anxiety frequency and the prediction of fearfulness Behav Res Ther 1986 8 1 8 10.1016/0005-7967(86)90143-9 3947307

[B35] Domschke K Stevens S Pfleiderer B Gerlach AL Interoceptive sensitivity in anxiety and anxiety disorders: an overview and integration of neurobiological findings Clin Psychol Rev 2010 8 1 11 10.1016/j.cpr.2009.08.008 19751958

[B36] Fukudo S Saito K Sagami Y Kanazawa M Can modulating corticotropin releasing hormone receptors alter visceral sensitivity? Gut 2006 8 146 148 10.1136/gut.2005.070888 16407379 PMC1856495

[B37] Kanazawa M Endo Y Whitehead WE Kano M Hongo M Fukudo S Patients and nonconsulters with irritable bowel syndrome reporting a parental history of bowel problems have more impaired psychological distress Dig Dis Sci 2004 8 1046 1053 15309899 10.1023/b:ddas.0000034570.52305.10

[B38] Tillisch K Mayer EA Pain perception in irritable bowel syndrome CNS Spectr 2005 8 877 882 16273016 10.1017/s1092852900019830

[B39] Gaylord SA Palsson OS Garland EL Faurot KR Coble RS Mann JD Frey W Leniek K Whitehead WE Mindfulness training reduces the severity of irritable bowel syndrome in women: results of a randomized controlled trial Am J Gastroenterol 2011 8 1677 1688 10.1038/ajg.2011.184 PMC6502251 21691341

[B40] Ljótsson B Falk L Vesterlund AW Hedman E Lindfors P Rück C Hursti T Andréewitch S Jansson L Lindefors N Andersson G Internet-delivered exposure and mindfulness based therapy for irritable bowel syndrome: a randomized controlled trial Behav Res Ther 2010 8 531 539 10.1016/j.brat.2010.03.003 20362976

